# Single-port robotic totally extraperitoneal inguinal hernia repair using a floating docking technique with the da Vinci SP system: feasibility across a spectrum of hernia complexity

**DOI:** 10.3389/jaws.2026.17632

**Published:** 2026-10-22

**Authors:** Junhyung Park, Seong-Taek Oh, Jaeim Lee

**Affiliations:** Department of Surgery, Catholic Kwandong University International St. Mary’s Hospital, Incheon, Republic of Korea

**Keywords:** da Vinci SP, floating docking, inguinal hernia, single-port surgery, totally extraperitoneal repair

## Abstract

The da Vinci SP system is applied to totally extraperitoneal (TEP) inguinal hernia repair, but clinical data remain limited. Full wrist articulation requires the instruments to extend beyond the cannula tip, which the preperitoneal space is too shallow to accommodate intracorporeally. We report a single-surgeon series using a floating docking configuration. We retrospectively reviewed 12 consecutive patients undergoing SP TEP repair. After a 2.5-cm infraumbilical incision and entry into the retrorectus space, the cannula was docked suspended approximately 9–10 cm above the skin, so that the instruments reached the operative field already articulating. Mesh reinforcement was performed in all patients. Patients were 11 men and 1 woman (91.7% male; mean age 57.2 ± 10.7 years), evenly split between sides; 10 primary and 2 (16.7%) recurrent, 8 indirect, 4 direct. Median operative and console times were 42.5 (IQR 38.8–46.8) and 40.0 min (IQR 33.8–42.2). All procedures were completed via a single port without conversion or additional trocars, including an incarcerated scrotal hernia and a recurrent hernia with dense adhesions requiring intracorporeal peritoneal closure. Mean hospital stay was 1.1 ± 0.5 days. Complications occurred in 3 patients (25.0%): fluid collection in all, urinary retention in one, managed conservatively. No recurrence occurred over a median follow-up of 6.9 months (IQR 4.1–8.7). SP TEP repair with floating docking was feasible across a range of hernia types and complexities, with no major early complications. Larger cohorts with longer follow-up are needed to assess recurrence and chronic pain.

## Introduction

Groin hernia repair is one of the most commonly performed operations worldwide, with more than 20 million procedures undertaken each year [1]. The totally extraperitoneal (TEP) approach is among the minimally invasive techniques widely used for this indication. To reduce the number of incisions and improve cosmesis, single-incision laparoscopic surgery has been applied to TEP repair, achieving perioperative outcomes and overall safety comparable to those of the conventional multi-incision approach [2, 3], with greater cosmetic satisfaction reported for the single-incision technique [3]; however, the single-incision technique is more challenging than the multi-incision approach [4] and has been associated with a longer operative time [3, 4].

To address these technical demands, robotic platforms have more recently been applied to inguinal hernia repair. Robotic repairs have most often been reported with the transabdominal preperitoneal (TAPP) approach, whereas reports of robotic TEP remain comparatively limited [5]. Because the TAPP approach requires entry into the peritoneal cavity, it carries a risk of intraperitoneal organ injury or intra-abdominal adhesion, which the extraperitoneal TEP route reduces [5]; robotic TEP is therefore an attractive alternative. It has indeed been performed through a single incision with the da Vinci Xi platform [5]. Being a multi-arm system, however, the Xi has several limitations when used through a single incision: its curved single-site instruments lack articulating joints, a recognised limitation [5], and their restricted range of motion and limited strength have hindered the wider adoption of this approach [6]; docking is also slower than with a dedicated single-port platform [6].

The da Vinci Single Port (SP) system (Intuitive Surgical, Sunnyvale, CA, USA) was developed specifically for single-port access. Delivering an articulating camera and three wristed instruments through a single arm, it minimises instrument collisions, improves dexterity, and offers a shorter learning curve than multi-arm systems [6], as well as greater instrument articulation [7].

Applying the SP platform to the TEP space nevertheless raises a specific constraint. The instruments must extend a minimum distance beyond the cannula tip before full wrist articulation becomes available [8], yet the beginning of the preperitoneal space is too shallow to accommodate that extension within the space. Single-port robotic TEP repair using this platform has been described as technically feasible [8], and interest in its use for inguinal hernia repair has grown in recent years. Nevertheless, clinical evidence remains limited and largely confined to early-experience reports: a stage 1 study under the IDEAL (Idea, Development, Exploration, Assessment, Long-term study) framework described the first European case series of da Vinci SP robotic hernia repair [9], and direct comparative data remain limited, with a multi-institutional, propensity score-matched study comparing the da Vinci SP platform with single-incision laparoscopy using an articulating instrument (ArtiSential) for TEP repair [10]. Accordingly, we report a single-surgeon, single-institution series in which the above constraint was addressed by a floating docking configuration maintained throughout most of the procedure, with a focus on feasibility and operative efficiency. Within the IDEAL framework, this work corresponds to stage 2a (development), in which a technique is refined and described in a consecutive series before comparative evaluation.

## Methods

This retrospective case series included 12 consecutive patients who underwent da Vinci SP-assisted TEP inguinal hernia repair performed by a single surgeon at one institution between August 2025 and May 2026. Patients with any type of inguinal hernia scheduled for elective repair were eligible; no patient was excluded based on hernia type, defect size, or prior surgical history. The primary outcome was operative (skin-to-skin) time, and secondary outcomes included console time, conversion rate, intraoperative complications, length of hospital stay, postoperative complications (graded according to the Clavien–Dindo classification), and recurrence during the follow-up period.

Follow-up was conducted by outpatient clinic visit. Inguinal fluid collection was diagnosed by the operating surgeon using bedside ultrasonography at these visits. Aspiration was performed only when the estimated volume of the collection exceeded 10 mL; no collection in the present series met this threshold, and all were therefore managed by observation alone and confirmed to have resolved by the 1-month outpatient visit. Recurrence was assessed by clinical examination at each visit; routine imaging was not performed. Continuous variables were expressed as mean ± standard deviation or median and interquartile range (IQR) where appropriate, and categorical variables were expressed as number and percentages. Since this study is a descriptive case series, inferential statistics were not applied. The manuscript was prepared in accordance with the STROBE statement for observational studies.

### Patient preparation and docking

The patient is placed in the lithotomy position with approximately 10° of Trendelenburg tilt. The robotic system is docked from the patient’s right side.

### Skin incision and rectus exposure

A 2.5-cm longitudinal skin incision is made just below the umbilicus. The anterior rectus sheath of the inguinal hernia side is transversely opened, and the rectus muscle is retracted laterally to expose the posterior rectus sheath.

### Port insertion, insufflation, and floating docking

After exposure of the posterior rectus sheath, a low-profile single-port access device with a soft patient-contact surface (the ONEPORT Tebah Korea, Inc., Ltd., Republic of Korea), is inserted into the retrorectus space, and carbon dioxide insufflation is initiated. The robotic cannula is docked using a floating technique, positioned approximately 9–10 cm above the skin surface (Figures 1A,B). The floating docking is essential during TEP inguinal hernia repair when using the da Vinci SP platform because robotic instruments must extend at least 9 cm beyond the tip of the cannula to achieve full wrist articulation [8]. The retrorectus and preperitoneal spaces are too shallow to accommodate this extension within the space, and articulation would therefore remain incomplete if the cannula were located at the abdominal wall level. Suspending the cannula above the abdominal wall allows the required extension to be taken up outside the body, so that the instruments reach full articulation before passing through the access device and entering the operative field. Unlike other commonly performed SP procedures, SP-TEP inguinal hernia repair typically requires maintenance of the floating docking configuration throughout most of the operation, with dynamic readjustment of the cannula height as the dissection proceeds.

**FIGURE 1:**
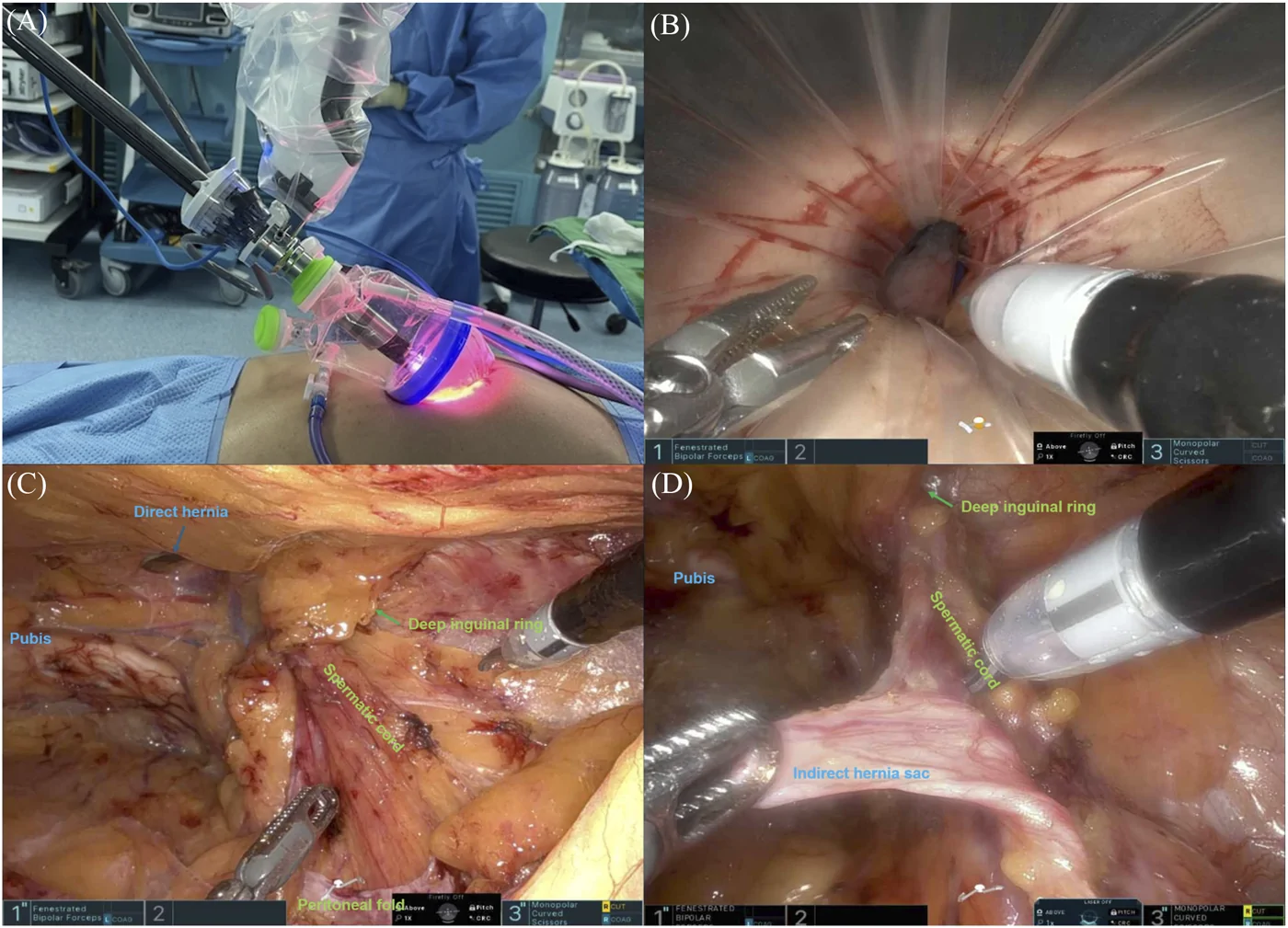
Intraoperative photographs of single-port robotic totally extraperitoneal inguinal hernia repair with the da Vinci SP system. **(A)** External view of the floating docking configuration, in which the robotic cannula is suspended approximately 9–10 cm above the skin surface without fixation to the abdominal wall, so that the extension required for full wrist articulation is taken up outside the body. **(B)** Endoscopic view obtained after the floating docking configuration has been established, showing the fenestrated bipolar forceps and the monopolar curved scissors above the skin incision site before they enter the retrorectus space; the extension required for wrist articulation is taken up over this segment, outside the abdominal wall. **(C)** Operative field in a direct hernia, showing the pubis, the direct hernia defect, the deep inguinal ring, the spermatic cord and the peritoneal fold after reduction. **(D)** Operative field in an indirect hernia, showing the indirect hernia sac dissected from the spermatic cord with the deep inguinal ring and pubis identified.

### Dissection of spaces and hernia sac identification

Two robotic instruments are used: a fenestrated bipolar forceps in the left arm and monopolar scissors in the right arm, with the endoscope positioned above the instruments (above mode). When necessary, an additional grasper is used. After the instruments are inserted, dissection is carried out along the retrorectus plane toward the extraperitoneal space. Similar to conventional laparoscopic TEP repair, the retropubic space is developed first, followed by the retroinguinal space. During this process, direct hernia sacs are identified and reduced (Figure 1C). In cases of indirect inguinal hernia, the spermatic cord structures are completely dissected and identified. The spermatic fascia is then opened to isolate the indirect hernia sac. The sac is ligated using a Hem-o-lok clip at the preperitoneal fat level, and the distal sac is excised (Figure 1D).

### Mesh application, fixation, and closure

A mesh is introduced into the developed extraperitoneal space by the patient-side assistant and positioned using a fenestrated bipolar grasper and monopolar scissors. After adequate deployment and coverage of the myopectineal orifice, the mesh is secured with fibrin glue. Following confirmation of appropriate mesh placement, desufflation is performed. The robotic system is then undocked, the anterior rectus sheath is closed, and the skin incision is sutured.

## Results

Between August 2025 and May 2026, 12 consecutive patients underwent da Vinci SP–assisted TEP inguinal hernia repair. The mean age was 57.2 ± 10.7 years, and the cohort was predominantly male (11 of 12, 91.7%). The mean body mass index was 24.9 ± 4.8 kg/m^2^. Hernias were equally distributed between the right and left sides (6 and 6, respectively), and all patients were classified as American Society of Anesthesiologists physical status II. Two patients (16.7%) presented with a recurrent hernia. The baseline demographic and clinical characteristics are summarised in Table 1.

**TABLE 1:** Baseline demographic and clinical characteristics of patients undergoing da Vinci SP–assisted totally extraperitoneal (TEP) inguinal hernia repair.

Variable	Total (n = 12)
Age (yr)	57.2 ± 10.7
Male sex, n (%)	11 (91.7)
BMI (kg/m^2^)	24.9 ± 4.8
Hernia location (R/L), n (%)	6/6 (50.0/50.0)
ASA PS (I/II/III), n (%)	0/12/0 (0/100.0/0)
Hx prostate cancer surgery, n (%)	0 (0)
Recurrent hernia, n (%)	2 (16.7)
Smoking, n (%)	3 (25.0)
BPH, n (%)	2 (16.7)
Hypertension, n (%)	6 (50.0)
Diabetes mellitus, n (%)	4 (33.3)

Age and BMI are presented as mean ± standard deviation, and categorical variables as number (%). ASA PS, american society of anesthesiologists physical status; BMI, body mass index; BPH, benign prostatic hyperplasia.

All procedures were completed as da Vinci SP–assisted TEP repairs, with no conversion to open surgery or to conventional laparoscopy and no additional trocars. The median operative (skin-to-skin) time was 42.5 min (interquartile range [IQR], 38.8–46.8), and the median console time was 40.0 min (IQR, 33.8–42.2). Robotic docking was rapid, completed within 1–2 min in all cases. Eight hernias were indirect (66.7%) and four were direct (33.3%), with no femoral hernias; the median defect size was 2.0 cm (IQR, 1.5–3.0). One patient (8.3%) had a large inguinoscrotal hernia and an incarcerated segment of small bowel, which was reduced and repaired without conversion. A 15 × 10 cm mesh was used in seven patients (58.3%) and a 13 × 9 cm mesh in five (41.7%). An intraoperative complication occurred (1 of 12, 8.3%): a peritoneal opening during repair of a recurrent hernia with dense adhesions, which was closed primarily by intracorporeal suturing without conversion. The remaining intraoperative outcomes are presented in Table 2.

**TABLE 2:** Perioperative outcomes of da Vinci SP–assisted TEP inguinal hernia repair.

Variable	Total (n = 12)
Intraoperative	
Op time (min)	42.5 [38.8–46.8]
Console time (min)	40.0 [33.8–42.2]
Hernia type (D/I/F), n (%)	4/8/0 (33.3/66.7/0)
Defect size (cm)	2.0 [1.5–3.0]
EHS classification (M2/L1/L2/L3), n (%)	4/6/1/1 (33.3/50.0/8.3/8.3)
Scrotal descent, n (%)	1 (8.3)
Incarceration, n (%)	1 (8.3)
Mesh 15 × 10/13 × 9, n (%)	7/5 (58.3/41.7)
Intraop complication, n (%)	1 (8.3)
Postoperative	
LOS (days)	1.1 ± 0.5
Overall complication, n (%)	3 (25.0)
Inguinal area fluid collection, n (%)	3 (25.0)
Skin wound seroma, n (%)	0 (0)
Urinary retention, n (%)	1 (8.3)
Conversion, n (%)	0 (0)
Clavien-Dindo I–II, n (%)	3 (25.0)
Clavien-Dindo III–IV, n (%)	0 (0)
Readmission 30d, n (%)	0 (0)
Reoperation 30d, n (%)	0 (0)
Recurrence, n (%)	0 (0)
Follow-up (months)	6.9 [4.1–8.7]

Operative time, the primary outcome, was measured as skin-to-skin duration. Operative time, console time, defect size, and follow-up duration are presented as median [interquartile range]; length of hospital stay is presented as mean ± standard deviation; and categorical variables are presented as number (%). For the EHS classification, M denotes a medial (direct) and L a lateral (indirect) hernia, with the accompanying number indicating defect size (1, ≤1.5 cm; 2, 1.5–3 cm; 3, >3 cm). Intraoperative complication denotes any complication occurring during surgery (here, one peritoneal opening during a recurrent-hernia repair). Overall complication denotes the number of patients with any complication, and complications were graded by the Clavien–Dindo classification; one patient had both fluid collection and urinary retention, so percentages reflect individual complication frequencies and sum to more than the overall complication rate. Inguinal area fluid collection was diagnosed by the operating surgeon using bedside ultrasonography; none required aspiration, and all had resolved by the 1-month outpatient visit. The single episode of urinary retention was managed with an alpha-blocker. Recurrence was assessed by clinical examination during the follow-up period. EHS, european hernia society; IQR, interquartile range; LOS, length of hospital stay; D, direct; I, indirect; F, femoral.

The mean length of hospital stay was 1.1 ± 0.5 days. Postoperative complications occurred in three patients (25.0%), all of which were Clavien-Dindo grade I or II. All three developed an inguinal-area fluid collection (25.0%); none reached the threshold for aspiration, and all had resolved with observation alone by the 1-month outpatient visit. One of these patients also experienced urinary retention (8.3%) that was managed with an alpha-blocker. No skin wound seroma, readmission, or reoperation occurred within 30 days. No recurrence occurred during a median follow-up of 6.9 months (IQR, 4.1–8.7). The postoperative outcomes and complications are detailed in Table 2.

## Discussion

In this single-institution series of 12 consecutive patients, da Vinci SP-assisted TEP inguinal hernia repair was completed in every case without conversion to open surgery or to conventional laparoscopy. Importantly, the cohort was not confined to small or simple defects: it comprised both indirect (eight) and direct (four) hernias and included both primary and recurrent (two) cases, while the European Hernia Society classification spanned several categories (M2, L1, L2, and L3), with defects of up to 3 cm. One patient had a large inguinoscrotal hernia containing an incarcerated segment of small bowel, and another underwent repair of a recurrent hernia with dense adhesions, during which a peritoneal opening occurred and was closed primarily by intracorporeal suturing without conversion. Taken together, these findings indicate that single-port robotic TEP repair can be performed successfully across a range of hernia types and anatomical complexities, extending earlier work that described the technique as feasible [8] and adding to the still-limited clinical evidence on this approach [9]. This is particularly relevant because the grasping force of single-port instruments has been regarded as a potential limitation in patients with dense adhesions or large hernia sacs [10]; in our experience, however, even such cases were completed without conversion.

The technical element that distinguishes this series is the floating docking configuration. Its rationale is geometric rather than incidental. Full wrist articulation requires the instruments to extend a minimum distance beyond the cannula tip [8]; this requirement is a fixed property of the instruments, whereas the preperitoneal space is too shallow to accommodate it. Rather than attempting to create that depth within the abdomen, floating docking relocates where the distance is spent: with the cannula suspended above the abdominal wall and unfixed to it, the required extension is traversed outside the body, and the instruments arrive at the operative field already articulating. This reframing is what makes the confined plane workable, and it also explains why the configuration must be maintained, and its height dynamically readjusted, through most of the operation rather than only at the outset, unlike SP procedures performed within a large cavity. It was applied in all cases.

The same distance can also be obtained by a dedicated accessory. The SP Access Port Kit provides an external chamber in which the instrument joints articulate outside the incision, supplying the required extension by a different route. We did not use it, for two reasons specific to TEP repair. Its overall volume is large, and the rigid plastic component that seats on the abdominal wall exerts considerable compression on the skin around the narrow infraumbilical incision used in this approach. We therefore selected a low-profile access device with a soft patient-contact surface (ONEPORT), already approved and in routine use for laparoscopic and single-port robotic surgery in Korea, and obtained the extension by suspending the cannula instead. Floating docking is therefore an accessory-independent alternative to the dedicated port rather than a replacement for it.

Although robotic transabdominal preperitoneal (TAPP) repair is currently more prevalent [5, 11], partly because the confined preperitoneal space makes the totally extraperitoneal (TEP) approach technically demanding [8, 10, 11], TEP offers a distinct technical advantage by entirely avoiding peritoneal cavity entry. Entry through the peritoneal cavity also obliges the surgeon to incise the peritoneum in order to reach the preperitoneal space and then to close it over the mesh at the end of the procedure. This additional step adds technical demand, since the closure must be achieved by intracorporeal suturing, and it represents an operative stage that the extraperitoneal route dispenses with altogether, since neither the opening nor the closure is required. While the confined preperitoneal space presents recognised technical challenges [8, 10], the present series demonstrates that the intrinsic advantages of the TEP approach can be successfully preserved. All procedures were executed via a single-port extraperitoneal approach, effectively managing complex cases including inguinoscrotal extension, incarceration, and prior abdominal surgery.

Within the extraperitoneal approach, the choice of robotic platform is also important. Single-incision robotic TEP was initially undertaken with the da Vinci Xi platform [5]. As a multi-arm system used through a single incision, however, the Xi relies on curved single-site instruments that lack articulating joints, a recognised limitation [5]; their restricted range of motion and limited strength contributed to the limited adoption of these single-site instruments [6], and docking is slower than with a dedicated single-port platform [6]. By contrast, the da Vinci SP system was purpose-built for single-port access, delivering a fully articulating camera and three wristed instruments through a single arm; this design minimises instrument collisions, improves intracorporeal dexterity [6, 10], and offers greater instrument articulation [7], together with a shorter learning curve and faster docking than the Xi [6]. Our experience aligns with the advantages of the SP platform. In all cases, robotic docking was completed within 1–2 min, reflecting the streamlined setup of the SP system, and the floating configuration was established without additional setup time.

The operative profile observed in this series further supports the operative efficiency of the single-port robotic approach. The median operative (skin-to-skin) time was 42.5 min and the median console time was 40.0 min, and the small difference between these two measures indicates that the non-console steps of the procedure—docking, undocking, and wound closure—added little time to the overall operation. The short and uniform operative times, together with the rapid and consistent docking described above, point to an efficient operative workflow.

The early postoperative course was likewise uneventful in this series. All postoperative complications were minor, corresponding to Clavien–Dindo grade I or II, and followed a self-limited course: the inguinal-area fluid collections resolved spontaneously, and the single episode of urinary retention was managed conservatively with an alpha-blocker. No skin wound seroma, readmission, or reoperation occurred within 30 days, and the mean hospital stay was 1.1 ± 0.5 days, consistent with an uneventful early recovery. The only intraoperative complication—a peritoneal opening encountered during repair of a recurrent hernia with dense adhesions—was closed primarily by intracorporeal suturing without conversion, illustrating that the articulating instruments of the SP platform can accommodate unexpected intraoperative events within the confined preperitoneal space.

This study has several limitations that should be considered alongside its findings. First, it is a retrospective, single-institution case series of 12 patients treated by a single surgeon, without a concurrent comparison group; accordingly, the sample size limits statistical precision, and the results are best regarded as hypothesis-generating. Second, as a single-centre, single-surgeon series, the findings may not be directly generalisable to other surgeons or institutions, and the number of cases is insufficient to characterise a learning curve. Third, the absence of a control group precludes direct comparison with conventional laparoscopic or other robotic techniques. Fourth, recurrence was assessed by clinical examination alone without routine imaging, so small asymptomatic recurrences may have been missed. Finally, a median follow-up of 6.9 months is sufficient to exclude early technical failure but remains too short to assess long-term recurrence, and no patient-reported outcome or quality-of-life instrument was applied to evaluate chronic postoperative pain. Larger, prospective, and ideally multicentre comparative studies with longer follow-up and standardised patient-reported outcomes will therefore help to define more precisely the role of single-port robotic TEP repair in inguinal hernia surgery.

In summary, da Vinci SP-assisted TEP inguinal hernia repair using a floating docking configuration is feasible across a range of hernia types and anatomical complexities, with no major early complications. The consistent single-port setup, short and uniform operative times, and uneventful early recovery further support the reproducibility of the procedure. Larger comparative studies with longer follow-up are warranted to confirm these findings, particularly regarding recurrence and chronic pain.

## Data Availability

The datasets presented in this article are not readily available because of institutional restrictions on the sharing of patient-level clinical data; de-identified data are available only on reasonable request and subject to institutional review board approval. Requests to access the datasets should be directed to JL, primalee96@ish.ac.kr.
